# Biologics for the treatment of severe asthma: Current status report 2023

**DOI:** 10.5578/tt.20239921

**Published:** 2023-06-13

**Authors:** G. Paçacı Çetin, S. Kepil Özdemir, Ö. Can Bostan, N. Öztop, Z. Çelebi Sözener, G. Karakaya, A. Gelincik Akkor, İ. Yılmaz, D. Mungan, S. Bavbek

**Affiliations:** 1 Division of Immunology and Allergy, Department of Chest Diseases, Erciyes University Faculty of Medicine, Kayseri, Türkiye; 2 Division of Allergy and Immunology, Department of Chest Diseases, University of Health Sciences, Dr. Suat Seren Chest Diseases and Surgery Training and Research Hospital, İzmir, Türkiye; 3 Division of Immunology and Allergy, Department of Chest Diseases, Hacettepe University Faculty of Medicine, Ankara, Türkiye; 4 Clinic of Adult Immunology and Allergy, Başakşehir Cam and Sakura City Hospital, İstanbul, Türkiye; 5 Clinic of Immunology and Allergy, Ankara Bilkent City Hospital, Ankara, Türkiye; 6 Division of Immunology and Allergic Diseases, Department of Internal Medicine, İstanbul University Faculty of Medicine, İstanbul, Türkiye; 7 Division of Immunology and Allergy, Department of Chest Diseases, Ankara University Faculty of Medicine, Ankara, Türkiye

**Keywords:** biologics, effectiveness, efficacy, safety, severe asthma

## Abstract

**ABSTRACT:**

**Biologics for the treatment of severe asthma: Current status report 2023:**

Severe asthma is associated with increased use of healthcare services, significant deterioration in the quality of life, and high disease and economic burden on patients and societies. Additional treatments are required for severe forms of asthma. Biological agents are recommended for the treatment of severe asthma. In this current status report, we aimed to evaluate the efficacy, effectiveness, and safety data of approved biologics; omalizumab, mepolizumab, reslizumab, benralizumab, dupilumab, and tezepelumab, in the treatment of severe asthma and appropriate patient profiles for these biologics. Pubmed and Cochrane databases based on randomized controlled trials, posthoc analyses, meta-analyses, and real-life studies examining the efficacy and effectiveness of biologics in severe asthma were searched, and the results of these studies on important asthma outcomes were reviewed. Existing studies have shown that all the approved biologic agents targeting cells, receptors, and mediators involved in type 2 inflammation in the bronchial wall in severe asthma significantly reduce asthma exacerbations, reduce the need for oral corticosteroids, and improve asthma control, quality of life, and pulmonary functions. Characterizing the asthma endotype and phenotype in patients with severe asthma and determining which treatment would be more appropriate for a particular patient is an essential step in personalized treatment.

## Abstract (tr)

**Ağır astımda biyolojik ajanların ilk seçiminden biyolojikler arasında geçişe kadar biyolojik tedavi yönetimi:**

Ağır astım; sağlık hizmeti kullanımında artış, yaşam kalitesinde önemli derecede bozulma ve hasta ve toplum üzerinde yüksek hastalık yükü ve ekonomik yük ile ilişkilidir. Ağır astım formları için ek tedaviler gereklidir. Biyolojik ajanlar ağır astımda önerilen son basamak seçeneklerdir. Bu güncel durum raporunda biyolojiklerin (omalizumab, mepolizumab, reslizumab, benralizumab, dupilumab, tezepelumab) ağır astımdaki etkinlik, etkililik ve güvenlilik verilerini ve bu biyolojikler için uygun hasta profillerini değerlendirmek amaçlanmıştır. Bu amaç doğrultusunda Pubmed ve Cochrane veri tabanlarında ağır astımda kullanım onayı almış biyolojiklerin ağır astımda etkisinin değerlendirildiği randomize kontrollü çalışmalar, post-hoc analizler, meta-analizler ve gerçek yaşam çalışmaları bulunmuş ve biyolojiklerin ağır astım üzerindeki etkilerini değerlendirmek için gözden geçirilmiştir. Mevcut çalışmalar bronş mukozasındaki tip 2 inflamasyonda rol alan hücreler, reseptörler ve medyatörleri hedefleyen biyolojiklerin tümünün astım ataklarını anlamlı şekilde azalttığını, oral kortikosteroid ihtiyacını azalttığını, astım kontrolü, yaşam kalitesi ve solunum fonksiyonlarında düzelme sağladığını göstermiştir. Ağır astımlı hastalarda astım endotipinin ve fenotipinin doğru belirlenmesi ve buna uygun biyolojik tedavinin seçilmesi kişiselleşmiş tedavi yaklaşımın temel noktasıdır.

The aim of this work is to develop a comprehensive guiding document on the indications and usage of biologic drugs commonly employed in the treatment of severe asthma, specifically for specialists involved in managing asthma. A working group, comprised of professionals with expertise in severe asthma from various medical centers, has initiated the process of creating a current status report focusing on the use of biologics in severe asthma. The group convened through webinars to establish the study’s methodology, content structure, and subsections. Five key questions were generated by the working group, and the article’s content was organized to address and incorporate the answers to these questions ( 1 ).

Question 1: What is severe and difficult to treat asthma?

Question 2: What is the pathogenesis of asthma?

Question 3: What is the concept of severe asthma phenotypes and endotypes?

Question 4: What are the biologics approved for severe asthma and what are their target molecules?

Question 5: Which biologics could be used according to the phenotype in severe asthma?

Subsequently, task groups were established for each approved biologic used in the treatment of severe asthma, and these groups meticulously documented comprehensive information about each specific biologic. To gather relevant data, thorough searches were conducted in the PubMed and Cochrane databases, focusing on randomized controlled trials (RCTs), post-hoc analyses, meta-analyses, and reallife trials that investigated the efficacy of biologics in severe asthma. Detailed tables were created for each subsection pertaining to individual biologics. A separate search was performed for each biologic using similar methods, terms, and filters. Presentation abstracts that lacked full-text articles, congress abstracts, and studies not published in English were excluded from the analysis ( Figure 1 ) ( 1 ).

**Figure 1:**
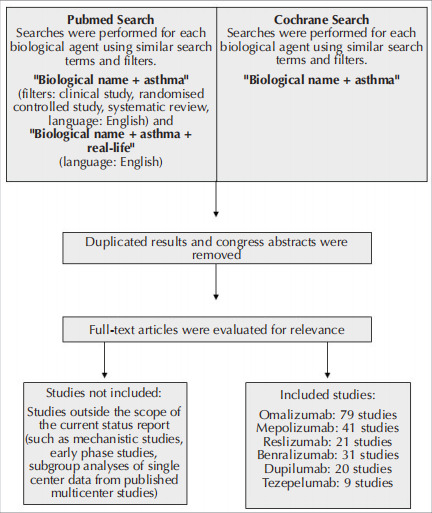
Flow chart for selection of the studies evaluated during the preparation of the current status report.

## INTRODUCTIN

Question 1: What is severe and difficult to treat asthma?

Asthma is a chronic lower airway disease with variable airflow limitation and accompanying airway inflammation. It is an umbrella diagnosis that includes complex pathophysiological mechanisms, inflammatory pathways, and variable clinical courses. It affects approximately 334 million people worldwide and this number is expected to approach 400 million in the near future (2,3).

Asthma, that is uncontrolled (poor symptom control or a history of at least two attacks per year requiring oral corticosteroid (OCS) use, or at least one asthma attack per year requiring hospitalization) despite moderate or high dose inhaled corticosteroid (ICS) treatment with a second controlling agent, often longacting beta-agonist (LABA), or maintenance OCS is defined as difficult asthma. Asthma control may be difficult due to inappropriate inhaler technique, poor treatment adherence, smoking or other comorbidities, or incorrect asthma diagnosis (3-5). Despite the provision of correct inhaler technique and adherence to the inhalers, control of deteriorating factors and triggers, patients who needed high dose ICS/LABA ± other controller agents to control the disease or remained uncontrolled were considered to have severe asthma (3,6).

Severe asthma, which affects 3.7-7% of all asthmatic patients, is associated with increased use of healthcare services, significant deterioration in the quality of life for both the patients and their families, and high disease and economic burden on societies (6). In our country, a single-center study reported that 7% of 300 adult patients with asthma were identified as having severe asthma based on the criteria set by the Global Initiative for Asthma (GINA). Additionally, a multicenter study conducted in tertiary care facilities found that the prevalence of severe asthma was determined to be 12% (7). While mild-to-moderate forms of asthma can often be effectively managed with available treatment options such as low-tomedium dose ICS and LABA, the management of severe asthma typically necessitates additional treatment modalities. At this point, accurate diagnosis, appropriate determination of the subtype of severe asthma, and identification of suitable candidates for specific biologic therapies are of utmost importance (3,8).

Question 2: What is the pathogenesis of asthma?

**Figure 2:**
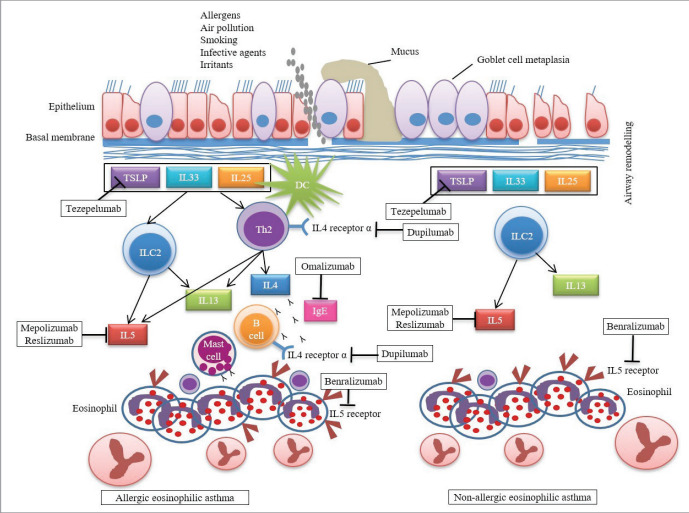
Type 2 inflammation pathways and biologicals targeting these pathways in asthma. Airway epithelial cells are stimulated to produce alarmins (IL25, IL33, TSLP) as a result of interactions with allergens and/or irritants and these in turn interact with cells of the innate and adaptive immune system to activate eosinophilic inflammation in allergic and non-allergic eosinophilic asthma endotypes. (DC: Dendritic cell, TSLP: Thymic stromal lymphopoietin; ILC2: Type 2 innate lymphoid cell; IL: Interleukin).

Persistent airway inflammation is an important feature of asthma. Inflammation is usually accompanied by an increase in airway smooth muscle mass, thickening of subepithelial lamina reticularis, matrix deposition in airway walls, an increase in microvessels and neural networks, and mucous metaplasia. Airway inflammation is a predominant event in asthma and is observed from the early stages of the disease. The intense inflammatory and immunological cell infiltration observed in the airways results from both activation of resident cells and the migration of inflammatory cells from the circulation to the airways. An important feature of the inflammatory reaction in asthma is its multicellularity. Different asthma phenotypes show different inflammatory characteristics. However, in most phenotypes, eosinophils constitute the main cellular component of inflammation in the airway walls and lumen. Neutrophils, lymphocytes, macrophages, mononuclear cells, and mast cells accompany inflammation to varying degrees in different asthma subtypes. Both innate and acquired immune system cells and epithelial cells play a role in the pathogenesis of asthma. Traditionally, asthma has been perceived as an eosinophilic disease characterized by the activation of Th2s, but type 2 innate lymphoid cells (ILC2s) and basophils can also initiate eosinophilic inflammation in patients with asthma. Although eosinophilic inflammation is characteristic of asthma, some patients do not have eosinophilic inflammation, consistent with the heterogeneity of asthma. Neutrophils are detected in some patients, particularly in smokers (9). The main role of T cells in asthma is the regulation of the allergic immune response with a strong Th2 cell response (type 2 response). However, other T cell types such as Th1, Th3, and Th17 are also detected in different asthma endotypes. T helper cells are stimulated by antigen-presenting dendritic cells, macrophages, and B cells. After stimulation of the T cell receptor with the presented antigen, helper T cells mature predominantly via Th2 or Th1 pathways. Local cytokine microenvironment and genetic tendencies play a determinative role in this differentiation. Airway hypersensitivity and bronchial smooth muscle contraction, which develop as a result of or related to inflammatory pathogenesis, cause a decrease in airway diameter. Generally, inflammation is accompanied by features of airway remodeling, such as submucosal fibrosis, bronchial smooth muscle hyperplasia, and hypertrophy, an increase in mucus-secreting cells, and changes in vascular and endothelial function (Figure 2) (1).

Question 3: What is the concept of severe asthma phenotypes and endotypes?

According to cluster analysis, five asthma phenotypes have been identified:

1- The early-onset mild atopic asthma,

2- Early-onset mild to moderate atopic asthma,

3- Early-onset severe atopic group,

4- Late-onset non-atopic eosinophilic asthma,

5- Late-onset non-atopic non-eosinophilic asthma (10-12).

In addition, the immune pathogenesis observed in the airways of individuals with asthma, referred to as endotypes, is studied by categorizing them into subgroups based on the profile of inflammatory cells and the mediators involved (11-13). In this context, two basic endotypes based on the predominant inflammatory cells have been defined in order to guide phenotypic/endotypic treatment approaches and to find appropriate treatment options. These are type 2 (T2) (T2 high) asthma and non-T2 (T2 low) asthma (13).

At least half of the patients with asthma have type 2 asthma, characterized by the involvement of T helper (Th2) lymphocytes, ILC2s (innate lymphoid cells type 2), mast cells, natural killer cells (NKs), and the release of cytokines such as IL-5, IL-4, and IL-13. This endotype also encompasses their receptors, specific immunoglobulin (Ig) E, mediators, and molecular components (3-5). In this endotype, eosinophilic cell infiltration of the bronchial wall, lower force expiratory volume in one second (FEV1) values, more bronchial hypersensitivity, more OCS use, increased emergency department admissions and increased asthma attacks, therefore more severe asthma clinic have been observed (12). Sputum and blood eosinophilia, serum-specific IgE levels, and fractional exhaled nitric oxide (FeNO) are noninvasive biomarkers that indicate the presence of type 2 inflammation. Within this endotype, there are subphenotypes known as allergic eosinophilic asthma and non-allergic eosinophilic asthma (14). Allergic asthma starts in childhood and usually persists into adulthood. In this particular group, cytokines such as IL-4, IL-13, and IL-5, along with cellular components including CD4 T lymphocytes, ILC2s, and mast cells, play significant roles. This type of asthma is more prevalent during childhood and adolescence, and individuals with year-round allergen sensitivity are more likely to carry it into adulthood (10,15). There is a positive correlation between total IgE levels and hospitalization due to asthma, as well as the requirement for higher doses of ICS. The monoclonal antibody omalizumab, which targets IgE, has been recognized as the preferred choice for patients with allergic asthma when high-dose ICS/LABA treatment is insufficient for disease control and a biologic agent is necessary (10,16). Another well-defined phenotype is nonallergic eosinophilic asthma which typically begins in the 40s-50s. There is an eosinophilic inflammation that is resistant to the treatment of corticosteroids. Asthma is difficult to control, and frequent attacks, frequent need for systemic steroids, and fixed airway obstruction occur at an early stage. Sinusitis, nasal polyposis, and sometimes nonsteroidal anti-inflammatory drug sensitization may accompany clinical features. In this particular subgroup, there is no elevation of IgE or allergen sensitivity. Instead, eosinophilic inflammation is driven by ILC2 lymphocytes and the release of IL-4, IL-5, and IL-13 from these cells, independent of allergen exposure (10,17,18).

The remaining half of patients with asthma have a non-type 2 asthma endotype characterized by the involvement of Th1 and Th17 cells, as well as cytokines released by these cells, including IL-17A and IL-17F. In this endotype, there is a presence of neutrophilic inflammation in the bronchial mucosa. The mechanisms underlying non-type 2 asthma are not yet fully understood, and these patients generally show less responsiveness to steroid treatment. Unfortunately, targeted therapies for this endotype present challenges at present. A subset of patients exhibits features of both endotypes, with the presence of both eosinophils and neutrophils. In another group of patients, no inflammatory cells are observed, which is referred to as paucigranulocytic inflammation (12).

Question 4: What are the biologics approved for severe asthma and what are their target molecules?

In recent years, the identification of phenotypes in severe asthma has facilitated the development and rapid approval of phenotype-specific biologic agents (16). Monoclonal antibodies (MoAbs), which are recommended as biologic agents for severe asthma, target the cells involved in the type 2 endotype, as well as the cytokines released from these cells and their receptors. This current status report provides detailed information on the approved biologics, namely omalizumab, mepolizumab, reslizumab, benralizumab, dupilumab, and tezepelumab, for the treatment of severe asthma (Table 1).

Question 5: Which biologics could be used according to the phenotype in severe asthma?

## Omalizumab

Omalizumab is a MoAb that binds to the constant region of free IgE in serum, preventing its interaction with high- and low-affinity IgE receptors, FcH RI and FcH RII, particularly on mast cells, basophils, and B lymphocytes (19). It reduces circulating IgE levels regardless of allergen specificity and inhibits IgE binding sites and hence activation of mast cells and release of inflammatory mediators (20). Therefore, it has been demonstrated to inhibit the allergic cascade and to be effective in the treatment of severe allergic asthma. Various RCTs, meta-analyses, and real-life studies reported that omalizumab treatment provides clinically and statistically significant reductions in asthma exacerbations, decreases the need for OCSs, improves asthma control, quality of life, and respiratory functions in patients with severe allergic asthma sensitized with at least one perennial allergen and uncontrolled despite the combination of medium-to-high dose ICS and LABA ± other controller therapy (21-27).

## Who are the candidates for omalizumab treatment?

Patients with severe asthma, whose symptoms cannot be controlled despite medium-to-high dose ICS + LABA ± other controller therapy.

≥6 years of age, body weight 20-150 kg, and sensitive to at least one perennial allergen confirmed by skin tests or specific IgE positivity, and serum total IgE level of 30-1500 IU/mL.

**Table 1:** MoAbs approved for use in severe asthma and other allergic/inflammatory diseases accompanying asthma, manufacturers, target molecules, doses, and indications

MoAb and brand name	Manufacturer FDA approval date	Target molecule	Dose/Route of administration	Indications (Step 5 treatment)
Omalizumab (XOLAIR^®^)	Genentech/Novartis Asthma= 2003 CSU= 2014 NP= 2020	IgE	SC 2-4 w Calculated based on weight and IgE level	Age ≥6 years, severe allergic asthma (in perennial allergen sensitive asthma) (in Türkiye, age ≥12 years) NP¶ ≥18 years CSU ≥12 years.
Mepolizumab (NUCALA^®^)	Glaxo Smith Kline Asthma= 2015 EGPA= 2017 HES= 2017 NP= 2021	IL-5	SC 100 mg/4 w	Age ≥6 years, severe eosinophilic asthma EGPA¶ ≥18 years
Reslizumab (CINQAIR^®^)^*^	Teva Pharmaceuticals 2016	IL-5	IV infusion 3 mg/kg/ 4 w	Age ≥18 years, severe eosinophilic asthma
Benralizumab (FASENRA^®^)^*^	Astra Zeneca 2017	IL-5Rα	SC Loading dose: First 3 doses 30 mg/4 w Followed by 30 mg/8 w	Age ≥12 years, severe eosinophilic asthma
Dupilumab (DUPIXENT^®^)	Regeneron Pharmaceuticals / Sanofi Genzyme Asthma= 2018 AD= 2017 NP= 2019 EE= 2022	IL-4RD (IL-4/IL-13)	SC a- Loading dose: 400 mg Followed by 200 mg/2 w b- Loading dose: 600 mg Followed by 300 mg/2 w	Age ≥12 years, a- Severe eosinophilic asthma b- Steroid dependent asthma AD ≥6 months ^ǂ^ NP^¶^ ≥18 years
Tezepelumab (TEZSPIRE^®^)	2021 Astra Zeneca/Amgen	TSLP	SC 210 mg/4 w	Age ≥12 years, severe asthma

MoAb: Monoclonal antibody, CSU: Chronic spontaneous urticaria, NP: Nasal polyposis, AD: Atopic dermatitis, HES: Hypereosinophilic syndrome, EGPA: Eosinophilic granulomatosis with polyangiitis, IgE: Immunoglobin E, IL-5: Interleukin 5, IL-5RD : Interleukin 5 receptor alpha, IL-4: Interleukin 4, IL-13: Interleukin 13, IL-4RD : Interleukin 4 receptor alpha, TSLP: Thymic stromal lymphopoietini, w: Week, IV: Intravenous, SC: Subcutaneous, EE: Eosinophilic esophagitis. * Not found in Türkiye. ǂ Approved in Türkiye ≥12 ages. ¶ Not approved in Türkiye for this indication.

### What are the response criteria?

Early-onset asthma, a significant association between symptom severity and exposure, a blood eosinophil level of ≥260 cells/µL, and a fractional exhaled nitric oxide (FeNO) level of ≥20 ppb have been identified as factors associated with a favorable response to omalizumab treatment.

### Mepolizumab

Mepolizumab is an IgG1/k class MoAb that inhibits the binding of IL-5 to its specific receptor, which causes eosinophils to mature in the bone marrow and migrate to the bronchial mucosa. It also acts by binding free IL-5. Consequently, eosinophilic airway inflammation is significantly reduced by inhibiting free IL-5 in both blood and sputum (28-30). In RCTs and real-life studies, treatment with mepolizumab has consistently demonstrated a statistically significant reduction in severe eosinophilic asthma exacerbations, a decrease in daily and/or exacerbation oral corticosteroid (OCS) requirements, improvement in asthma control, and enhancement in quality of life among patients with severe eosinophilic asthma who had not achieved adequate control despite receiving a high-dose ICS + LABA combination (28-37).

### Who are the candidates for mepolizumab treatment?

Patients with severe eosinophilic asthma, whose asthma cannot be controlled despite moderatehigh dose ICS + LABA ± other controller therapy,

Regardless of BMI, presence of atopy, or high serum IgE, mepolizumab treatment is effective in exacerbations and disease control in severe eosinophilic asthma.

### What are the response criteria?

Peripheral blood eosinophil counts ≥150 cells/ µL at the beginning of treatment or ≥300 cells/µL in the last year, ≥2 asthma attacks in the last year, presence of nasal polyposis and OCS dependence are found to be associated with better response to mepolizumab.

### Reslizumab

Reslizumab is an IgG4N humanized MoAb against IL-5. It inhibits the activity of eosinophils by binding to high affinity to IL-5, which promotes maturation, activation, survival, migration from the bloodstream, and entry into the airways, reduces the production of eosinophils and shortens their life span (38-40). In various RCTs and real-life studies, it has been reported that reslizumab treatment decreased asthma exacerbations, decreased daily or during the exacerbation OCS requirement, and improved asthma control in patients with severe eosinophilic asthma not adequately controlled despite medium/ high dose ICS + LABA combination (38-43). After reslizumab iv infusion, patients should be observed for 30 minutes, as anaphylaxis developed in 0.3% of patients in placebo-controlled studies (44).

### Who are the candidates for reslizumab treatment?

Uncontrolled severe eosinophilic asthma despite medium/high dose ICS + LABA ± any other controllers

### What are the response criteria?

Eosinophil >400 cells/µL, ≥2 asthma exacerbations in the last year, OCS-dependence, comorbid with nasal polyposis is associated with better response to reslizumab.

### Benralizumab

Benralizumab is a humanized MoAb directed against IL-5 receptor D. Benralizumab binds to IL-5 receptor D (IL-5RD ) on eosinophils, eosinophilic precursors, and basophils, thus preventing IL-5 binding to the receptor and causing rapid apoptosis of these cells through antibody-dependent cytotoxicity (45). It causes direct, rapid, and near complete depletion of eosinophils through antibody-dependent cellmediated cytotoxicity (46). Benralizumab treatment has shown efficacy in RCTs (45-47).

In both RCTs and several real-life studies, benralizumab has consistently demonstrated its efficacy in reducing asthma exacerbation rates and hospitalizations, decreasing the requirement for OCSs, improving asthma control, enhancing quality of life, and improving lung function in patients with uncontrolled, severe eosinophilic asthma, despite their use of high-dose ICS + LABA therapy (45-51). Meta-analyses have reported significant reductions in asthma exacerbations, improvements in quality of life, and increases in FEV1 with benralizumab treatment (52-54).

### Who are the candidates for benralizumab treatment?

Patients with severe eosinophilic asthma, in whom adequate asthma control cannot be achieved despite high-dose ICS + LABA ± other controller therapy constitute the appropriate patient group for benralizumab treatment.

Benefits of treatment increase with higher baseline rates of exacerbations and higher baseline blood eosinophil counts.

### What are the response criteria?

Blood eosinophil count of ≥300 cells/µL, ≥3 exacerbations in the last one year, use of OCS, presence of nasal polyposis, and age ≥18 at the time of diagnosis of asthma are factors associated with a better response to benralizumab.

### Dupilumab

Dupilumab is a human MoAb that specifically targets the IL-4 receptor-D , thereby inhibiting the signaling of both IL-4 and IL-13 (55). It has demonstrated efficacy in asthma, atopic dermatitis, eosinophilic esophagitis, and chronic rhinosinusitis with nasal polyposis. For the treatment of moderate-to-severe asthma, dupilumab is typically administered as a loading dose of either 400 mg or 600 mg, followed by a maintenance dose of 200 mg or 300 mg every other week.

Several RCTs and meta-analyses have shown that dupilumab reduced asthma attacks, improved symptoms, asthma control questionnaire (ACQ) and quality of life (AQLQ) scores, reduced the OCS dose by 70% and led to an increase in FEV1 with improved pulmonary functions in patients with uncontrolled asthma (56,57). Although asthma control, quality of life, and FEV1 were improved, and the use of rescue medication was reduced, dupilumab did not surpass the threshold of the minimal important difference (MID) in certain studies (58). However, in the subgroup with high blood eosinophils and high FeNO, the improvement in FEV1 was above the MID threshold. Results of the real-life studies are in line with the previous phase studies and meta-analyses (59-61).

### Who are the candidates for dupilumab treatment?

Patients with severe eosinophilic asthma in whom adequate control cannot be achieved despite high-dose ICS and LABA combination,

Patients with OCS-dependent severe asthma (eosinophil count and FeNO do not need to be high),

Patients with basal blood eosinophils ≥150 cells/ µL and ≤1500 cells/µL or FeNO ≥25 ppb, or requirement for maintenance OCS,

Patients with more than a specified number of severe exacerbations in the last year.

Severe asthma with moderate to severe atopic dermatitis and chronic rhinosinusitis with nasal polyposis.

### What are the response criteria?

Blood eosinophil count ≥300 cells/µL, experiencing more than one exacerbation in the past year, FEV_1_1.75 L, and elevated fractional exhaled nitric oxide (FeNO) levels have been associated with a favorable response to dupilumab treatment.

Patients with more severe asthma and higher T2 inflammation are good responders.

### Tezepelumab

The bronchial epithelium has gained considerable interest because of its role in the promotion and regulation of bronchial inflammation through the production of cytokines, including IL-25, IL-33, and thymic stromal lymphopoietin (TSLP). Among them, TSLP has been extensively studied as a therapeutic target in patients with severe asthma because it is involved in both type 2-high and type 2-low inflammation (62,63). Tezepelumab is a human MoAb specifically targeting TSLP (62,63).

The safety and efficacy of tezepelumab were evaluated in patients with uncontrolled asthma, despite treatment with a LABA and medium-to-high doses of ICS and LABA. The study demonstrated that tezepelumab significantly reduced asthma exacerbations by up to 71% compared to placebo, regardless of baseline blood eosinophil count, FeNO level, IL-5, IL-13, and periostin (64). Although another study did not observe a significant improvement in reducing OCS dose with tezepelumab compared to placebo, an improvement was observed in participants with baseline blood eosinophil counts of at least 150 cells per µL (65).

Tezepelumab was approved by FDA and by the EU as an add-on maintenance treatment for patients aged ≥12 years with severe asthma. It is the only biologic approved for severe asthma with no phenotype (e.g. eosinophilic or allergic) or biomarker limitations (66).

### Who are the candidates for tezepelumab treatment?

May be considered as a first-line biological agent in patients with poorly controlled, moderate-tosevere asthma, regardless of asthma phenotypes.

### What are the response criteria?

Patients with basal blood eosinophils ≥150 cells/ µL and higher FeNO are associated with better response to tezepelumab.

## CONCLUSION

Biological agents are effective targeted add-on treatments for severe asthma that cannot be controlled despite a maximum and effective dose of standard asthma treatment. Existing studies have shown that all of these agents targeting cells, receptors, and mediators involved in type 2 inflammation in severe asthma can significantly reduce asthma exacerbations, reduce the need for OCS, and improve asthma control, quality of life, and respiratory functions. Omalizumab, which targets circulating IgE, and the IL5-targeting agents; mepolizumab, benralizumab, and reslizumab, and most recently the epithelial-cell- derived cytokine, TSLP-targeting agent tezepelumab, are the biologics that have been currently approved for severe asthma and whose efficacy has been demonstrated in RCTs and/or real-life studies. Additionally, studies on new biological agents targeting type 2-high and type 2-low inflammation in severe asthma are in progress.

In conclusion, characterizing the asthma endotype and phenotype in patients with severe asthma and determining which treatment would be more appropriate for a particular patient is an essential step in personalized medicine. Current biological agents are leading clinicians to more individualized treatment plans for severe asthmatic patients.
